# Traces of the Invisible: How an Alternative Reading of *The Sleeping Beauty* Fashioned a Bookwork Heightening Awareness of the Role of the Anesthetist

**DOI:** 10.1007/s10912-019-09597-3

**Published:** 2020-01-14

**Authors:** Julie Brixey-Williams

**Affiliations:** 1 The Royal Society of Sculptors, London, UK; 2 University of Reading, Reading, UK

**Keywords:** Artist’s book, Anesthetics, Fairy tales, Visibility, Calligraphic trace

## Abstract

This article discusses a Leverhulme residency undertaken by the author Julie Brixey-Williams in 2003–4 at the Association of Anaesthetists of Great Britain and Ireland. Notions of medical visibility were explored through practice-led investigations under the umbrella title, *Traces of the Invisible*, that concentrated on making concrete, visible responses to the hidden or intangible elements of the anesthetist’s working life in areas such as sleep, breath, pain and genetic markers. *Rosebud* is a unique nine-foot concertina bookwork created after reading the entire story of *The Sleeping Beauty* into an anesthetic machine. This essay expands upon the concepts and material responses that led to the making of the book with particular reference to how the book’s structure forms a relationship to language and the body-as-site, whilst operating as a sculptural object that raises the visibility of the anesthetic profession. Fairy tales and their telling, including stories of enchanted sleep, transformational qualities, magical languages and shaman healers, will be examined alongside.

## Introduction

**Fig. 1:**
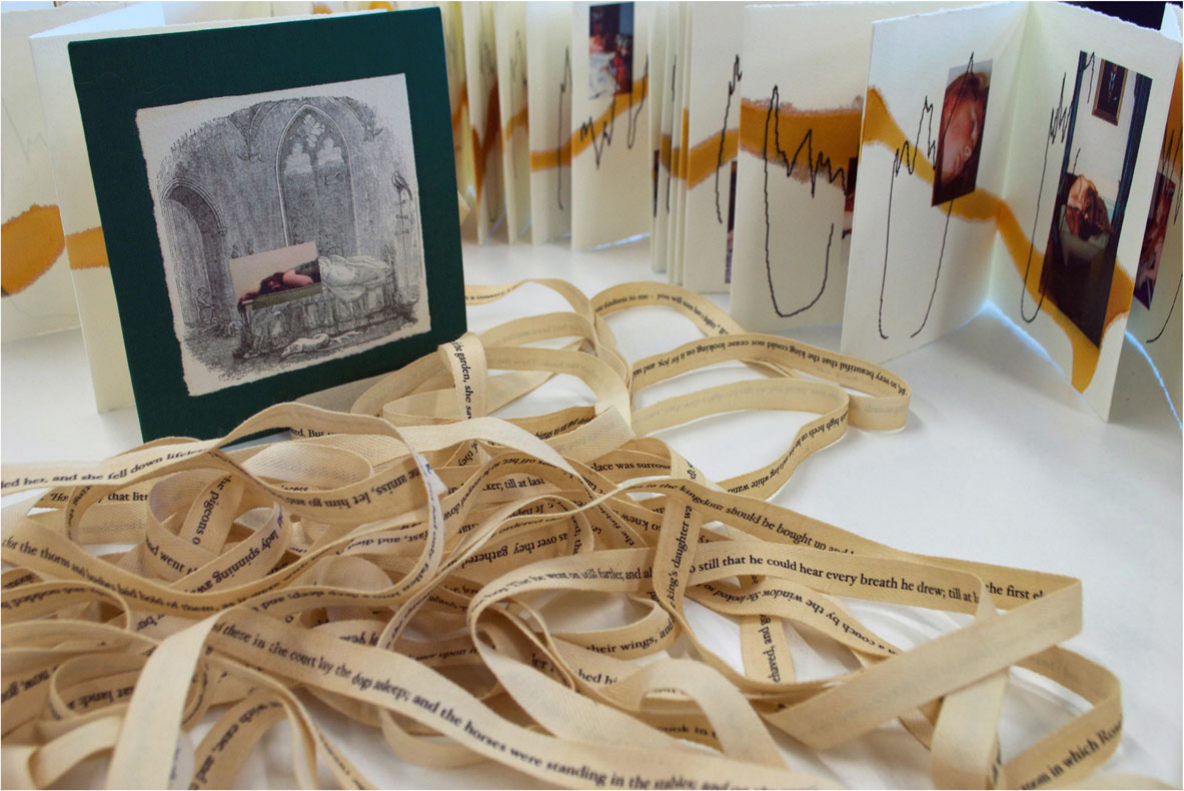
*Rosebud* (2005) original artist’s book showing green bookcloth cover, concertina structure, ribbon text and pages displaying flow loop waveforms and images © Julie Brixey-Williams

*Rosebud*, an original artist’s bookwork, comprises an entire reading of the tale of *The Sleeping Beauty* into an anesthetic machine and was created in collaboration with Dr. Anil Wijetunge at Central Middlesex Hospital, London, and fully completed in 2005 (Fig. 1). Assuming the role of both artist and reflector, I hope to reciprocally speak of, and listen to, the artwork in order to revisit the making process and look at the material physicality of its features. I will reflect on how these choices operate to open a conversation within the arena of medical humanities to heighten the visibility of an often hidden profession and to engage with the book’s unique architecture, which operates spatially between the covers to reflect the anesthetic journey. There will be a special focus on the relationship of the artist’s book with embodied practice, calligraphic trace and with the sleeping breathing body. I will suggest that choosing the form of a fairy tale opens poetic dialogue, and thus imaginative possibilities, within this exploration.

## The Leverhulme Residency

The Association of Anaesthetists of Great Britain and Ireland (hitherto referred to as AAGBI) was set up in 1932 to represent the medical and political views of its members. Their motto is *in somno securitas* (safe in sleep). The residency was the first of its kind for the AAGBI and was funded after a joint application to the Leverhulme Trust by Trish Willis, the curator of The Heritage Anaesthesia Museum, and myself. Residencies exist to invite artists, curators or academics from various disciplines to spend time with an organization or in an environment away from their usual practice arena. This provides time and space for reflection, new connections and relationships, questioning and often original perspectives and illumination. In this particular case, the aim was to heighten awareness of the anesthetic profession by fostering collaborative links between medical staff within hospitals, the administrative headquarters in Portland Place, London, and the Association’s Heritage Anaesthetic Museum. Assisting this investigation was a strong personal connection, as before studying fine art, I had worked for 14 years as an Intensive Care Respiratory Physiotherapist. Working in a team headed by anesthetists during that career had allowed me to develop a unique knowledge and understanding of their medical role, and yet I hoped that viewing with fresh eyes, from a distance of 10 years, would be a fruitful experience.

As a profession, anesthetists are relatively unseen, appear elusive, and the public often is unaware of the breadth of their work. After initial investigations to gather a historical perspective which included observing the operation of the AAGBI headquarters, drawing, reading and handling anesthetic equipment in the museum archive, I chose to concentrate on making concrete, visible responses to the hidden or intangible elements of the anesthetist’s working life in areas such as sleep, breath, pain and genetic markers. These elements chimed well with my own sculptural practice, which explores the dialogue between the body and lived space interpreted through performance and calligraphic trace to produce sculptural responses. In total, over a period of 10 months, eight individual projects addressing various aspects of anesthetic practice were completed in collaboration with different hospital departments across London.

## Scoping the terrain

The experimental French novelist Georges Perec (1997) states: “This is how space begins, with words only, signs traced on the blank page” (13). My process started with a series of thoughts about traces and drawings made in clinical practice, questioning the spaces that anesthetists and patients inhabit (corporeally and architecturally) and the quality of the relationship that the body shares with the equipment, particularly as one experiences a loss of consciousness. As part of my artistic practice, I often poach and re-contextualize approaches from different disciplines re-siting them from one sphere into another: a tactic of investigation I term “misreading.” Adopting this interactive, playful, somewhat subversive approach opens the doors of possibility and seemed one that suited a profession whose very role in the intensive care unit, for instance, is to find creative solutions to extremely ill patients who have, up to that point, confounded standard clinical approaches. A truly successful collaboration relies on making time, meeting in the middle to share knowledge, listening and then forging ahead into the unknown. We started on our journey.

**Fig. 2:**
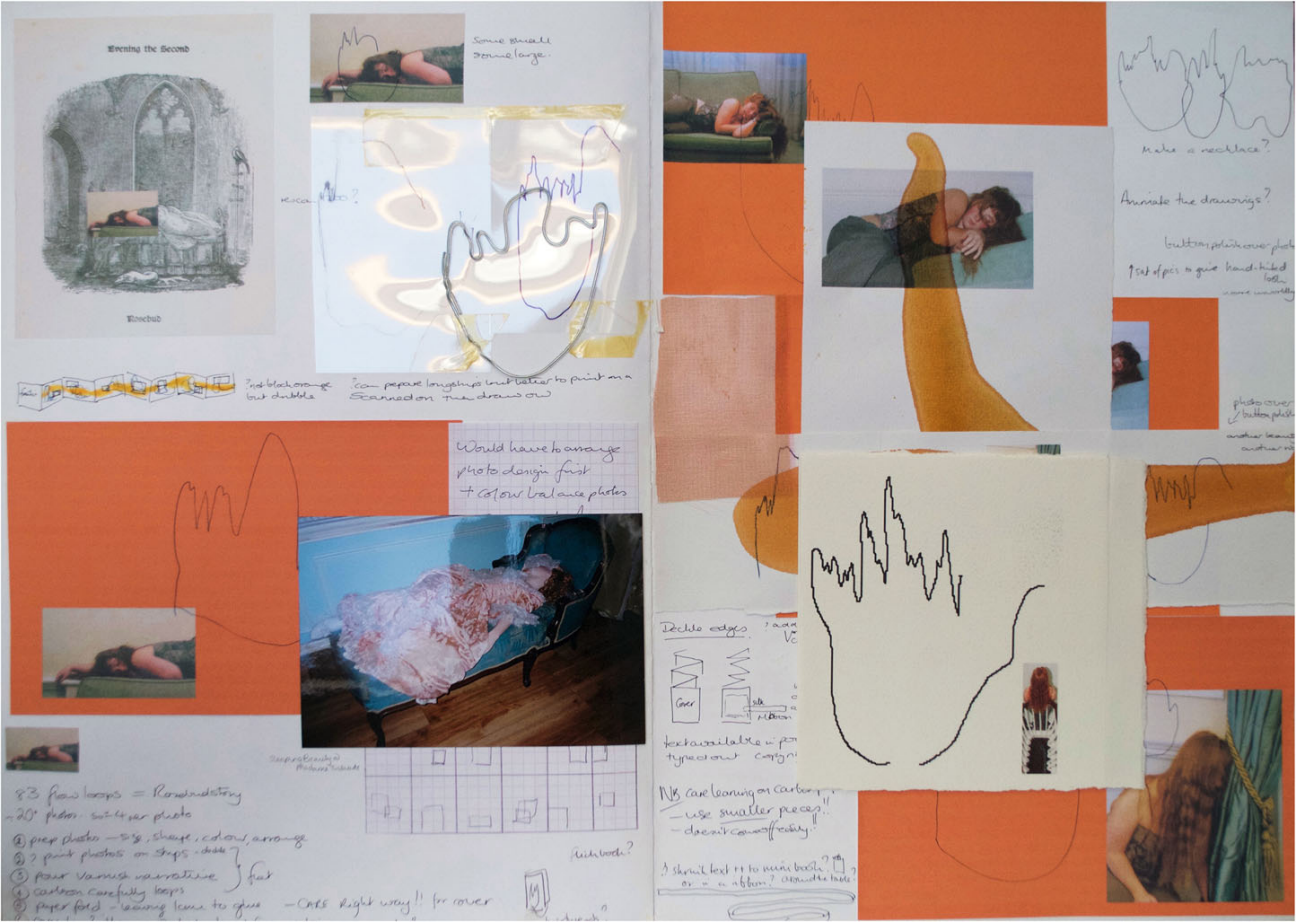
Sketchbook pages showing working process © Julie Brixey-Williams

A function was discovered on the 55 Anesthesia Monitor ADU Care Station that drew a flow loop diagram reflecting pressure changes in the status of an anesthetized patient’s inspiratory and expiratory phases of breathing. A series of playful experiments took place in pockets of time snatched between patients where Dr. Wijetunge and I spoke or sang into the machine. We compiled a list of words to perform relating to the practice of anesthetics: “anesthetist”; “sleep”; “inspiration”; “expiration” and “invisible.” (The shape of these single loops can be seen in the sketchbook pages in Fig. 2.) These flow loop characters created entirely by the gestural act of speech reminded me of calligraphy practice, and I first translated them into large, single glyphic paintings using Chinese ink. As Johanna Drucker (1998) describes: “The gestural mark is the trace of the very act of production as dynamic action” (65). Here my physical act of reading out loud was leading directly to the unfolding of hitherto unseen inscription. We had discovered a new medical language.

As the hieroglyphs magically unfolded on both paper scroll and light oscilloscope, it put me in mind of the oral tradition of fairy tales: by nature a fluid medium, taking on endless permutations, the personality of the teller and moving between the written and the spoken word (Warner 2014, 45). Performing an extended reading would additionally explore the effects of emotion and intonation upon the breath, and the creation of a sculptural book would give me scope to be playful with scale to enhance visibility, together with an unfixed, mutable form, thus introducing the unexpected or transformational as a means to engage the imagination. As a sufferer of sleep paralysis myself (defined as an intermediate state where a person is mentally conscious yet unable to move physically), the story of *The Sleeping Beauty* had always had resonance for me, plus I enjoyed the irony of a heroine in an enchanted sleep induced by a needle. A version of the tale, *Rosebud,* was selected from an Edwardian volume, *Gammer Grethel’s Fairy Tales* (undated, 31–36) for its length—9 min—and because it contained a wide vocabulary relating to the act of sleeping. The book’s connection with tales at bedtime used to lull children to sleep through the hypnotic effect of the voice, also seemed pertinent.

## Embodying the machine

To reduce any stress or liability to incoming patients, the recording of the work was completed with the co-operation of representatives at Datex Ohmeda in Hatfield, who generously provided a room, duplicate machine and projector. As the story was read, meticulous video recordings were made of the machine “writing” the flow loop diagrams as projections on a screen, which opened a space for analysis in the future, rather than during my emotional engagement with the script. Speech unfolded as a living, visible testament where the machine’s writing extended the reach of the body creating “a physical by-product, a material trace of human activity,” where speech was “birthed by the biological organs” and mediated by external tools, allowing the ephemeral to become permanent (Lupton and Miller 1999, 50). Invisible ether was fashioned by the voice and machine into a medium with shape and form, turning the laboratory into a poetic space.

Breathing is a universal act, and one that we do not consciously notice unless there is a lack of breath. Spoken language is usually produced during expiration, provided enough breath is present, and the need to inspire produces pauses. By choosing to focus my attention on an act that goes unnoticed but which requires skill, diagnosis and monitoring by anesthetists during surgery, I hoped to make breath itself the visible entity highlighting their work. It would be narrow, however, to describe this as the total range of the anesthetist’s role, and during the residency, I looked at many other areas such as pain management in chronic conditions and during elective Caesarian, plus a range of other mark-making activities. For example, one of these projects utilized the hand-drawn V-shaped symbols (colloquially known as *seagull*s) that record the patient’s blood pressure. Anesthetists at Southend General Hospital kindly gave me access to their operation charts so I could superimpose their *seagulls* onto a series of seaside postcards of the area.

In the operating theater, a seal would normally be formed between the anesthetized patient’s airway and the machine by the insertion of a cuffed tube in the trachea through the vocal cords. So certain adjustments were necessary to prevent air loss and allow enough pressure: namely a standard inflatable rubber facemask, connected by corrugated tubing to the machine held firmly against my mouth. Kreider (2014), drawing on De Certeau, suggests that “spaces of reading and writing are produced through embodied spatial practices, physically acting out or performing the material aspects of a particular spatial order or place,” and this mask adaptation served to emphasize a site-specificity between my act of performing the language and the medical equipment (68; De Certeau 1988, 117). It created an embodied space of reading and reception that was uniquely corporeal, similar to oral tradition where over history, each teller marks the tale, using their voice as a unique instrument and with the ability to add personal interpretation (Warner 2014, 77). The mask’s physical restriction, in truth, meant very little sound was able to escape whilst at the same time it imposed a slightly unnatural speech pattern (the equipment leaving its trace upon my voice) due to the emphatic articulation needed to create enough pressure to elicit the flow loop, leading to lightheadedness. The result of this was that the act of reading was a near silent rendition with a state of altered consciousness: over the course of time I was slowly transformed into Sleeping Beauty.

## Sculpting the space of the book

At the time of recording, I had not planned exactly what kind of container my book would be for the material generated. I even continued to consider alternatives: for example, a room installation involving an enormous embroidered eiderdown. As the performance rolled out longitudinally across the screen, it highlighted the passage of time, and this influenced my choice of a long concertina structure, which could create a sculptural book-as-object able to function differently in a variety of environments. This mutability of form where each opening and repositioning of the book creates a unique experience for the viewer also struck me as similar to the retelling of fairy tales. A transformational quality would also parallel the many leaps of faith necessary in tales where a hundred years may pass in a few pages and highlight the altered perception of the passage of time under anesthetic, which may collapse, expand or “crease” the perception in a similar way. The physical action of opening and closing the book is also reminiscent of the AAGBI archive’s early twentieth century bellows ventilators, as they rose and fell on inspiration and expiration.

*Rosebud*’s extended nine-foot length of 83 pages allows entry at many points with leaps across space (it may be viewed aerially as a whole as well as page by page), thus slowing down one’s reading. Michael Archer (1998) makes the link between lived space and narrative, suggesting “space…is nothing without the stories that bring it to life” (6). Taking its lead from the genre of fairy tales, *Rosebud* operates as a metaphorical object (from the Greek *metaphorai:* things which carry one across space) where the machine-created calligraphic signs on the paper facilitate the transportation of “the physical body outside of itself,” so it may be examined anew (De Certeau 1988, 115). It becomes a mixed space, allowing interplay and reinterpretation (Lefebvre 1998, 203).

As an object, a book is received and handled by the physical body, and the language within publishing is rife with bodily reference. For example, we refer to the “spine,” “body of the text,” the “appendix” or “glossary,” and place “footnotes” and “headers,” all of which function as “organs of the text” (Lupton and Miller 1999, 50–51). The next section will expand on each of the book’s features, and how they operate to produce a multi-layered encounter for the reader/viewer. (I will use “reader” when referring to written or spoken language or calligraphic line and “viewer” in reference to the book as sculptural object).

*Rosebud* has a hand-made cover with a digitally manipulated image of the original Cruickshank lithograph superimposed with my own photography, mounted and indented onto green bookcloth. The specifics of the images in the book will be discussed later in the article. The volume when collapsed is compact with a nostalgic hand-feel; the scale of book a child might take to bed. Soft pulpy Waterford paper allowed me to create a soft deckle edge to echo the paper in the antique volume that contained the original text for *Rosebud.* Commonly, during that period, books were bound with the pages folded and unopened (*intonso*), and trimming was required to allow the pages to be turned. Referencing the hand in this act of cutting subtly builds a link with surgery and strengthens the connection with the actions of the body. The 83 pages were folded, assembled, and glued by hand. There is a play between the artist’s hand and mechanical reproduction in the recreation of the loop drawings, which were manually traced directly off the video recording so that stringent accuracy was maintained but then digitally reproduced via Photoshop and printed onto paper. The loops do not create a continuous line but instead are parcels of sounds created by sentences or phrases. At the time, I reflected that this collection of signs could be regarded as a form of visual notation or score with the potential to be taken forward into another discipline, such as dance. The coherence and syntax relies on the gestural breath, like the flow of movement in calligraphy where the movement of forming counts (Ingold 2007, 135). As these hieroglyphs invest the paper landscape, their rhythm is punctuated by expanses of white space – pauses created by my personal response to the text – that accentuate the repetitive pattern of breathing in and out (Lefebvre 1998, 206). In calligraphic practice, white space is seen as a vitalizing counterpoint to line. Christine Flint Sato, a contemporary calligrapher, describes it as having an equal importance to the gestural mark because “the more vital the line, the more the white is energized” (2001, 24). However, an alternative reading might be considered in *Rosebud,* where these material areas of emptiness could be interpreted as symbolizing silent gaps in consciousness or sleep.

Trust is an important element of language, which is often taken as an instrument of veracity (Lefebvre 1998, 138). In *Rosebud*, although at first sight the trace might appear to be asemic (non-semantic) text, there is a direct, meaningful correlation of the calligraphic form to the spoken script. My speaking role may therefore be seen as artist-as-shaman, and the reader must suspend understanding until all is revealed. Shamanism has a strong link to medicine where traditionally spells and incantations are used to cure illness, divine the hidden or to act as intermediary between the real and the spirit worlds. Marina Warner states that spells are principally verbal and “act to deepen a sense that invisible forces exist around us” (2014, 30). In this case, those forces would be the ministrations of the anesthetist who works silently and unseen during surgery and where the administration of anesthetic is akin to falling under an enchantment. In addition, by mediating between a child’s tale and the reader, I aim to pierce the impenetrable barrier characterized by an expert’s medical language by moving the discourse into the familiar (De Certeau 1988, 7). According to Warner, using the fairy tale genre to communicate scientific knowledge was not unknown in the Victorian era (2014, 109).

**Fig. 3:**
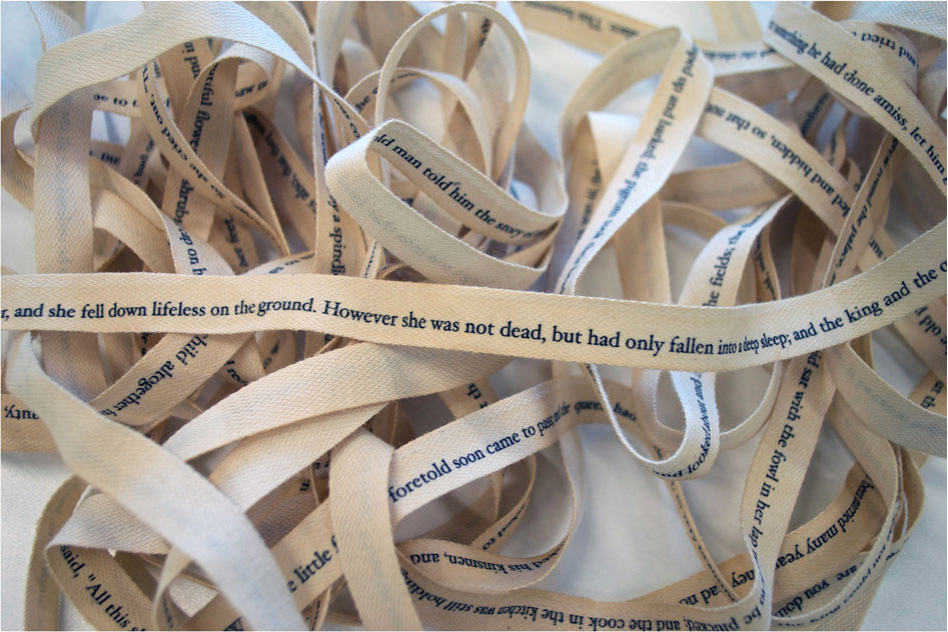
Ribbon text © Julie Brixey-Williams

Taking my cue from this idea of incantation, I created a very long off-white ribbon, upon was printed the entire version of the story in brown Hoefler Text typeface. Forming a continuous loop with the front and back covers and mimicking the flow loops themselves, it winds and falls in a variety of ways and may be used to wrap and close the volume. This sculptural motif acts as a counterpoint to the pages, opening a new space across and between to extend the time readers spend with the book as they try to reconcile the familiar story they hear as a voice in their own head with the rise and fall of the calligraphy that cannot be understood. They are searching for “the external sign of an internal recognition” (Merleau-Ponty 2003, 205). On the anesthetic machine, there is a certain lag (hysteresis) between the physical property of breathing and the changes visible on the oscilloscope. As the ribbon falls, tangles and undulates, the words jumble, slip and slide. It has its own time, rhythm and (literally) meter, and its disordered, non-linear, dysphasic form creates a palimpsest that puts one in mind of words overheard as one slips in and out of consciousness. As it responds to surface, place and gravity, certain words may appear on top, some become buried to defy sense, and random elements of the story may appear nearer the end or beginning. Visually, there is interplay between reading and viewing as the extreme sculptural length interacts with the sentences laid end-to-end, each element marking the passage of 100 years (Fig. 3).

Up until this point I have referred only to the inscription of line through the pages. There are however two further important visual elements in *Rosebud* that interact with the calligraphic forms: a continuous sweep of reddish varnish undulating from the front cover to the back and a series of images which begin at the exact moment Rosebud falls lifeless to the ground and disappear when the text refers to her awakening. These elements signify another important layer of visibility.

During the residency, research was published by Dr. Edwin Liem (2004) outlining the discovery of the first genetic marker for anesthetic requirement. From results using inhalational anesthetic, he stated that: “Red hair is the first visible human trait or phenotype” and concluded that: “redheads, on average require 20% more anesthetic to dull pain and obtain satisfactory sedation” (279). I decided to weave this important conclusion into my making, by adding a glossy shellac line that cut through the entire narrative like an oversized thick lock of red hair. Seeping, it enmeshes itself into the material structure of the paper, glossily catching the light and enlivening the delicate calligraphic black lines on the surface. The word “fairy” is linked to the Latin *fata* coming from the notion of the three Fates spinning and cutting the thread of prophecy (Warner 1995, 15). An alternative reading therefore could suggest that Rosebud cannot escape pricking her finger on the spindle, as it is embedded in the very fabric of her existence, in much the same way that redheads cannot influence their response to the needle that administers anesthetic.

There is so much to be said about blonde hair as a common magical trope in fairy tales that Warner devotes two chapters to the subject in *From the Beast to the Blonde*, but red is usually associated with being highly spirited, passionate, sexualized or as signifying sorcery (1995, 253–286). In *Rosebud*, however, it becomes a sign of quiet vulnerability and highlights a frightening thought that the patient might awake under paralysis, so I decided to include images in the book to speak directly to the anesthetists themselves.

**Fig. 4:**
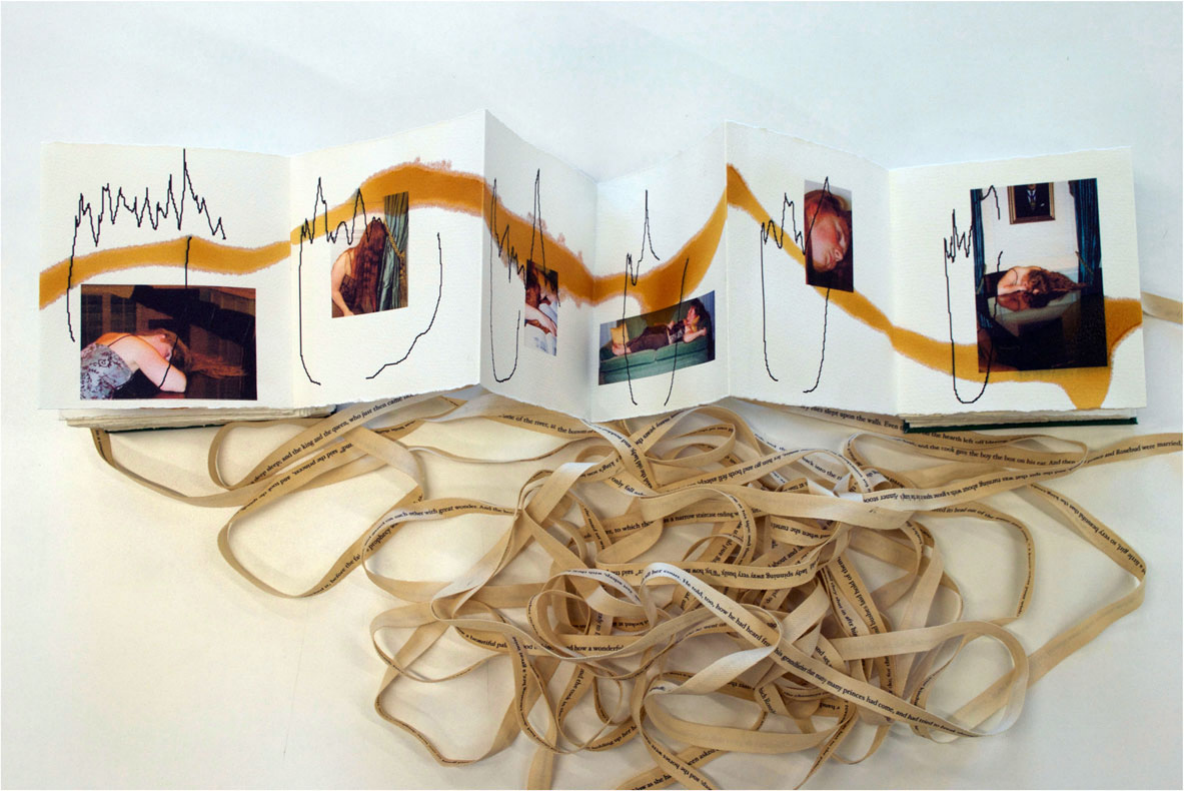
Book pages showing Alexa sleeping at various locations within the AAGBI, shellac line and ribbon © Julie Brixey-Williams

I advertised for a model with long red hair of either gender to be photographed at the AAGBI headquarters at Portland Place, London. I chose Alexa Reid, a drama student (with a particular interest in fairy stories and site-based work) with whom I felt I could have an open and experimental collaboration. Rather than wishing to illustrate the story, I wanted to position her sleeping figure within the seat of power of the AAGBI; thus placing her, as red-haired Rosebud, firmly back into the historical authority of the anesthetists’ space. She was literally in their power. She infiltrated the architecture: draping her hair over the winding staircase; sleeping on the long polished boardroom table; dozing over books in the library and snoozing across the furniture (Fig. 4). (One of the photographs against a marble fireplace with mirror later became a second piece of work in its own right. Her double image was digitally manipulated so one side was pale, the other rosy and perfused and then over-printed with Liem’s abstract as an invitation to look once, look twice).

As I sifted through the photographs, it stirred an early memory of being transfixed as a child by the mechanically breathing Sleeping Beauty waxwork at Madame Tussauds, London. Made by Curtis in 1767 (and apparently based on Louis XVs mistress, Madame Du Barry), it is believed to be the oldest waxwork in the collection. With her real red (amazingly) hair and eyelashes, she follows the tradition of the anatomical Venus of the eighteenth and nineteenth centuries. These idealized, often eroticized, supine figures were common in medical teaching, so using Alexa’s sleeping image as a teaching model seemed apt. As such though, it could be problematic as Alexa would be presented as a figure to be gazed upon like the Venus in a glass case, so I was very careful to give her the autonomy to choose her own poses, and she wore a top and trousers from her personal wardrobe. There is something very intimate about watching someone sleep. In 1995, Cornelia Parker created *The Maybe* with Tilda Swinton, where the actress slept in a glass case as a living sculpture at the Serpentine Gallery. Visitors could never be quite sure if she was truly asleep or merely day dreaming, but it gave them permission to watch. In the story, Rosebud has no one to watch her sleep until her deliverer arrives, but under anesthetic, we are vulnerable and have to trust that the anesthetist, as trained observer, will be watchful in order to monitor and adjust parameters to ensure our wellbeing.

## How *Rosebud* continues to articulate

To end, I would like to discuss how the book is recreated in each place it is presented and the role of the viewer’s engagement with it as an object. With its mutable form and many layers, the architecture between the covers is open to multiple readings. The acts of inscription in *Rosebud* exist in several “scriptural spaces” (Kreider 2014, 67). These include: the voice pressure changes creating trace within the machine; the oscilloscope projection that unrolled in front of the eyes; hand-written translation from screen to trace; digital printing onto the pages and the addition of the continuous shellac line. Alongside these acts of writing, the book creates a slippery sculptural space where its site-responsive structure reacts to surfaces and materials, and where meaning may be influenced by context. It is difficult to fix like the very nature of consciousness.

**Fig. 5:**
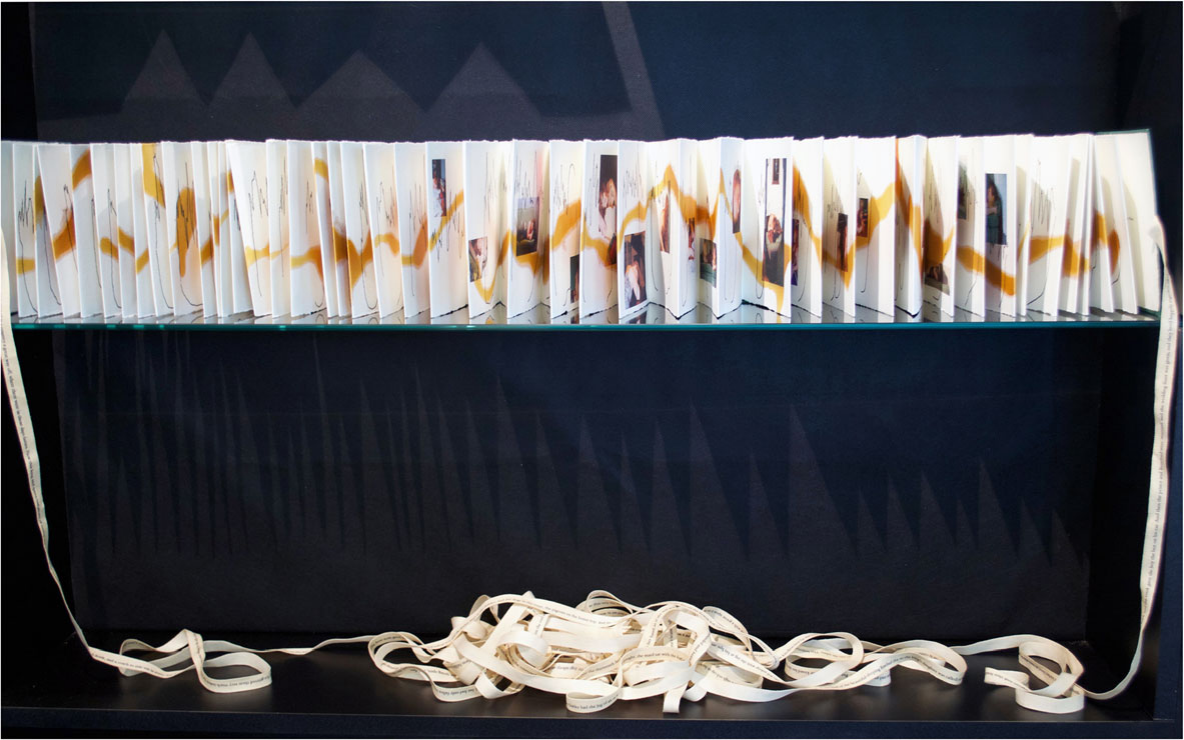
Glass case display at the Templeman Gallery, University of Kent, with lighting creating zigzag shadows. © Julie Brixey-Williams

It was first exhibited at an AAGBI function at Portland Place (comprising committee members, staff and invited guests including other doctors with whom I had collaborated) fully stretched out with the ribbon extending all around its curved surface on the outsized boardroom table. *Rosebud* once again took her repose on the surface but this time was watched by the very anesthetists whose role it was to ensure she was *in somno securitas.* Another time, the book was displayed as longitudinal scroll dwarfing the viewer who was forced to stretch their gaze up and down, and most recently, over two shelves in a glass case as befitting an anatomical Venus. In this last exhibition, at the Templeman Gallery, University of Kent, the ribbon lay on the shelf below the pages, softly tangled in undecipherable meaning, as if asleep. Linked to the pages on the shelf above by its attachments to the front and back covers, the ribbon forced the eye up and around in a continuous loop. Meanwhile, angled lighting caught the edges of the pages creating a series of menacing, sharp spindle-like shadows under the glass shelf, which added to its supernatural quality. (Fig. 5) Sayre (1989) speaks of the artist demanding a critical performance with the recipient needing to “fill in the gaps,” and I consider that at each presentation *Rosebud*’s audience is required to become viewer, reader and performer (250). This interplay creates slippage, allowing each person’s “lived experience” of the book, when situated in different sites, to add to its meaning (Lefebvre 1998, 203).

The configuration of the room at the AAGBI and the shape of the boardroom table, for instance, suggested that anesthetists perform a walk around in a complete circle, and they were invited to handle the pages and ribbon, move in and out to view or change the book’s sculptural configuration. Despite being able to recognize the flow loop forms, they were still required to enter the fairy tale and use their imagination to make sense of the elusive calligraphy. Used to reading charts and traces, some of the anesthetists picked up the notion of a score by relating physically to “the rhythm, tempo and thrust of the body” by mimicking the rise and fall of the lines and taking those amplitudes into their chests (Kreider 2014, 151). They literally embodied the text. As the gap between the unreadable glyphic traces and the jumbled text was bridged, each person became a storyteller. The unique voice in their head also became implicated in the piece; something Roland Barthes (1977) refers to as the “grain” so the book entered both the reader’s private physical and conscious space (188). In the crowded room, there were multiple silent stories being told.

Birthed in the very space of anesthetic practice and articulated through their equipment, *Rosebud* grew out of the spoken word and is planted firmly in the field of medical humanities. This story of enchanted sleep fulfills the flow and contraflow of oral tradition by starting with one version of a familiar fairy story that is transformed into an alternative magical, medical language to become a new rendition that demands to be passed on (Warner 2014, 72). Playfulness and an open mind during the collaboration allowed the anesthetic machine to become a space of questioning and poetic imagination, piercing the barrier of invisibility. Articulating space in a multi-formed way, the bookwork *Rosebud* focuses attention upon the work of the anesthetist by visually embodying the hidden acts of sleeping, breathing and speaking. Continually shifting, its multiple entry points, dynamic transformational structure and outsized dimensions facilitate leaps of faith, whilst demanding attention and trust from the viewer, just as each individual patient must when they place themselves in the hands of an anesthetist.
